# Pyrite nanoparticles as a Fenton-like reagent for in situ remediation of organic pollutants

**DOI:** 10.3762/bjnano.5.97

**Published:** 2014-06-16

**Authors:** Carolina Gil-Lozano, Elisabeth Losa-Adams, Alfonso F.-Dávila, Luis Gago-Duport

**Affiliations:** 1 Departamento de Geociencias Marinas, Universidad de Vigo, Lagoas Marcosende, 36310-Vigo, Spain; 2 Carl Sagan Center, SETI Institute, 189 Bernardo Avenue, Suite 100, Mountain View, CA 94043, USA

**Keywords:** copper phthalocyanine, Fenton-like reagent, hydrogen peroxide, nanoparticles, pyrite

## Abstract

The Fenton reaction is the most widely used advanced oxidation process (AOP) for wastewater treatment. This study reports on the use of pyrite nanoparticles and microparticles as Fenton reagents for the oxidative degradation of copper phthalocyanine (CuPc) as a representative contaminant. Upon oxidative dissolution in water, pyrite (FeS_2_) particles can generate H_2_O_2_ at their surface while simultaneously promoting recycling of Fe^3+^ into Fe^2+^ and vice versa. Pyrite nanoparticles were synthesized by the hot injection method. The use of a high concentration of precursors gave individual nanoparticles (diameter: 20 nm) with broader crystallinity at the outer interfaces, providing a greater number of surface defects, which is advantageous for generating H_2_O_2_. Batch reactions were run to monitor the kinetics of CuPc degradation in real time and the amount of H_2_O_2_. A markedly greater degradation of CuPc was achieved with nanoparticles as compared to microparticles: at low loadings (0.08 mg/L) and 20 h reaction time, the former enabled 60% CuPc removal, whereas the latter enabled only 7% removal. These results confirm that the use of low concentrations of synthetic nanoparticles can be a cost effective alternative to conventional Fenton procedures for use in wastewater treatment, avoiding the potential risks caused by the release of heavy metals upon dissolution of natural pyrites.

## Introduction

There has been growing interest in nanomaterials for green environmental remediation. For example, catalytically active synthetic nanoparticles inspired by natural minerals have been combined with in situ advanced oxidation processes (AOPs) as a potential strategy to remediate contaminants [1–3]. These AOPs generate hydroxyl radicals (OH^•^) that trigger the formation of other reactive intermediates (e.g., HO_2_^•^ and O_2_^•−^). Due to their high oxidation potential (*E*^0^ = 2.8 V), hydroxyl radicals attack most organic pollutants with rate constants in the order of 10^6^ to 10^9^ M^−1^·s^−1^ [4–5]. In practice, the formation of OH^•^ to degrade organic compounds involves the iron (Fe^2+^ and Fe^3+^) catalysed decomposition of H_2_O_2_; this transformation is known as the Fenton reaction [6]. A major drawback of conventional Fenton chemistry for wastewater treatment is that it requires a continuous supply of H_2_O_2_ under strict pH control, to limit the precipitation of iron oxyhydroxides. However, this control makes the process difficult and cost expensive.

Green nanotechnology can be used in various industrial and in situ remediation processes and can be an effective option for wastewater treatment. Several studies have recently reported the capacity of mineral suspensions (e.g., silicates, oxides and sulfides) to continuously generate H_2_O_2_ at surface defect sites [7–14]. Since the catalytic performance of the particles depends on the surface area-to-volume ratio (i.e., better performance is enabled by higher ratios), nanoparticles are typically expected to be more reactive than microparticles. Following this logic, and given the fact that pyrite microparticles have already been explored in Fenton chemistry [15], we sought to explore pyrite nanoparticles as heterogeneous catalysts for Fenton-like systems. Pyrite, the most abundant iron sulfide in the crust of Earth, releases Fe^2+^ and H^+^ upon oxidative dissolution. As such, it has been used with H_2_O_2_ as a Fenton catalyst for the degradation of several organic pollutants, including trinitrotoluene, carbon tetrachloride and diclofenac [16–19]. Several recent studies have reported that pyrite can spontaneously produce H_2_O_2_ via catalytic dissociation of O_2_ and H_2_O with iron surface sites [7–8,10,12–14]. Albeit the use of pyrite alone as Fenton reagent (i.e., without externally added H_2_O_2_) has been demonstrated for lactate degradation [15], this approach remains poorly studied and, to the best of our knowledge, has never been used to remove dyes from textile wastewater.

In this work, we compared synthetic pyrite nanoparticles to naturally derived pyrite microparticles for their efficiency in the oxidative degradation of copper phthalocyanine (CuPc), a representative dye that has a metallic ring structure. Phthalocyanines are widely used in the textile industry and can provoke carcinogenesis. However, removal of metallocyanines is difficult because of their resistance towards oxidative degradation as well as their low biodegradability. To this end, we performed real time experiments to determine the degradation rate of CuPc induced by H_2_O_2_ generated at the surface of the pyrite nanoparticles or microparticles, using UV–vis adsorption spectroscopy and H_2_O_2_ sensors.

## Results

### Structural aspects of the pyrite nanoparticles

**Figure 1:**
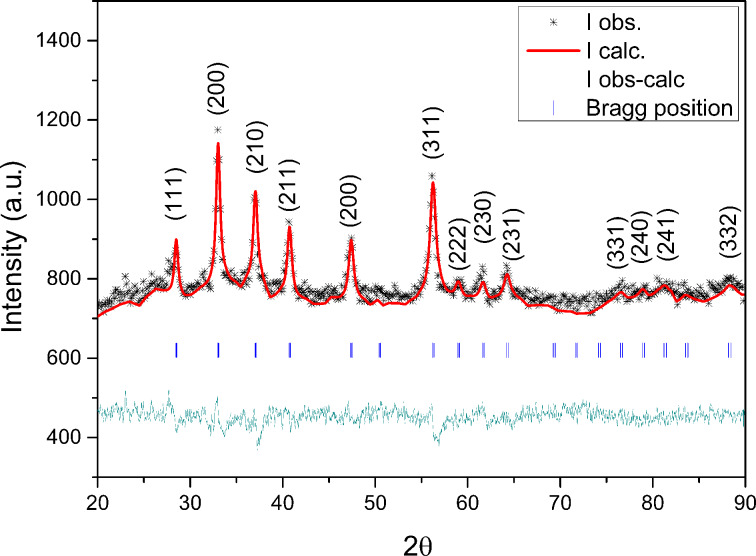
XRD pattern of the nanoparticles, indicating that pyrite was the only crystalline phase resulting from the synthesis. Rietveld analysis of the reflection broadening gave an average crystallite size of 20 nm.

The pyrite nanoparticles were analyzed at bulk level by XRD to evaluate the possible formation of secondary phases (Figure 1). As shown in the XRD pattern, the only observable Bragg reflections correspond to lattice planes of the cubic structure of pyrite (JCPDS card no. 42-1340). A moderate amount of background is also present in the pattern, where it is especially marked at the tail of the intensity distribution. This type of broadening is a characteristic indicator that coherent X-ray diffraction is occurring in finite-size domains (e.g., sub-grains). The average size of the crystalline domains (as calculated by Rietveld analysis) was 20 nm, which is consistent with the HR-TEM observation of the individual particles.

**Figure 2:**
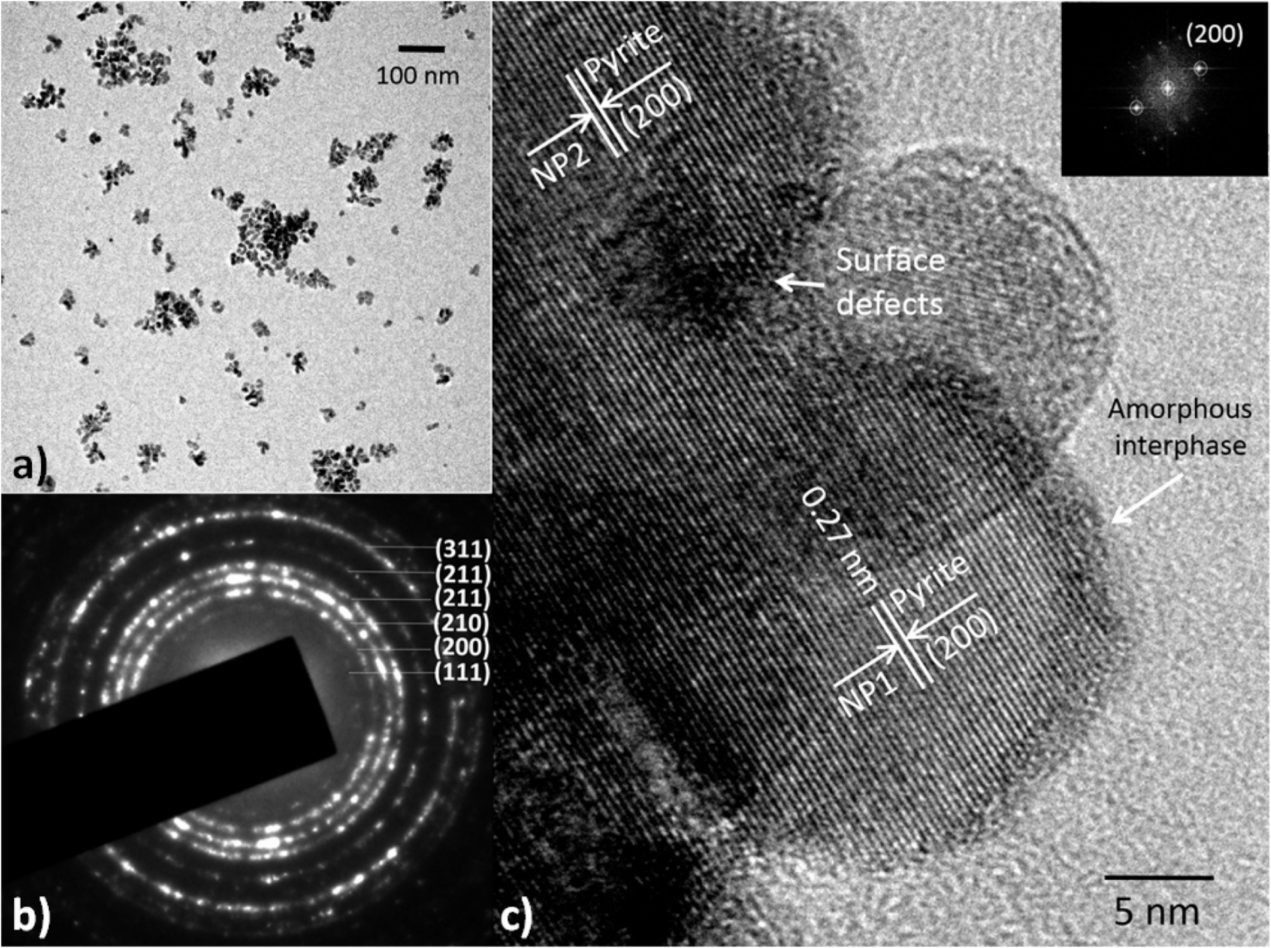
a) TEM image of the typical distribution of the nanoparticles, comprising polycrystalline aggregates of pyrite. b) SAED pattern of the nanoparticles. c) HR-TEM image showing the structure of the nanoparticles. Individual nanoparticles tended to self-assemble, thereby forming finite-size crystalline domains extended over several particles. The surface of the nanoparticles always appeared to be surrounded by an amorphous layer.

The TEM micrographs of the nanoparticles are shown in Figure 2. The upper left panel (Figure 2a) corresponds to a low-magnification image that shows the typical arrangement of nanocrystals obtained in our syntheses. The nanoparticles tended to aggregate, forming polydisperse clusters of rounded particles ranging in size from 20 nm (individual nanoparticle) to 150 nm (largest cluster). At the initial stages of the process, the clusters were polycrystalline, as indicated by the SAED pattern (Figure 2b). However, as crystallization progressed, some of the particles tended to reorganize, giving rise to single-crystal domains that extended to several particles. This feature is observable in the HR-TEM image (Figure 2c), in which two particles have self-assembled and their lattice fringes, corresponding to the (200) planes, exhibit coherent interference domains. This fact is also confirmed by the FFT superposition of the two particles (inset), which reveals that the diffraction spots have identical orientation. Interestingly, individual particles were surrounded by an amorphous layer (thickness: ca. 2 to 3 nm), suggesting that crystallization had begun upon nucleation of an amorphous precursor and subsequently followed some type of structural reorganization associated with high-energy surfaces between adjacent particles [20–22]. Similar textures have been observed in other syntheses, especially when high concentrations of precursors were employed [23–26]. This factor can be a limitation when high crystallinity is required; in such cases, use of low reagent concentrations or surfactants is often required to keep the particles apart. However, for the purposes of our work, the formation of interphases with numerous defects is advantageous, since the generation of H_2_O_2_ relies precisely on the presence of these defect sites.

### Kinetics of H_2_O_2_ generation and CuPc decoloration

#### Effect of pyrite particle surface area on reactivity

**Figure 3:**
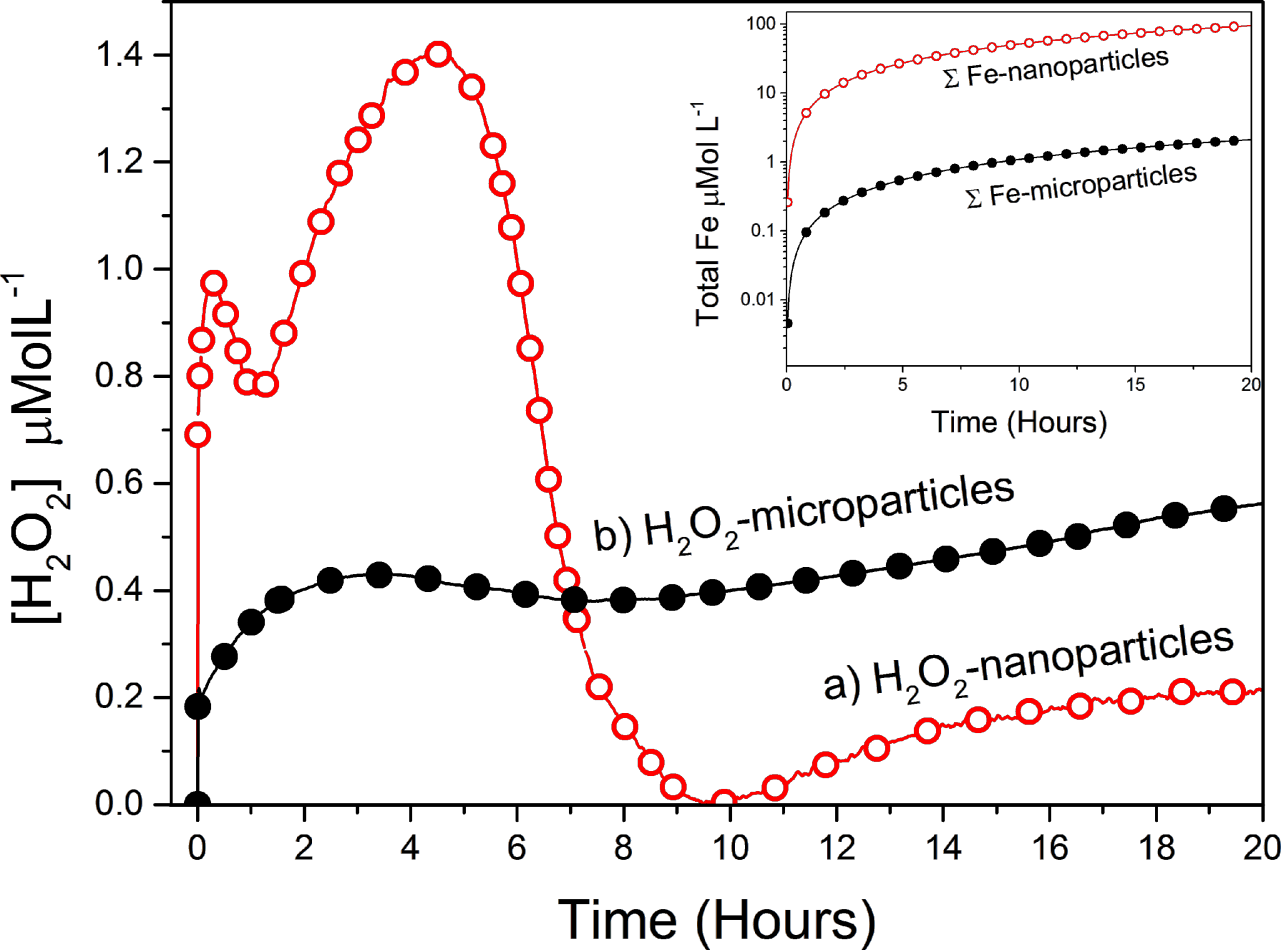
Plot of H_2_O_2_ concentration over time for suspensions of: a) pyrite nanoparticles or b) pyrite microparticles, at loadings of 0.08 g/L. Inset: Plot of PHREEQC calculations of total iron [Fe^2+^ + Fe^3+^] delivered in each case, assuming cubes of 20 nm for nanoparticles and 1.4 µm for microparticles.

In order to evaluate the influence of the pyrite particle surface area on the amount of H_2_O_2_ generated and on the dye decoloration (degradation) pathway, we performed kinetic experiments with dispersions of pyrite nanoparticles or microparticles (Figure 3, see section Experimental below). The same initial particle loading (0.08 g/L) and dye concentration (0.1 mg/L) were used. When nanoparticles were employed (Figure 3, curve a), the amount of H_2_O_2_ detected by the sensor oscillated, rose to a maximum value of 1.4 μM, rapidly decreased to zero and finally, plateaued at 0.2 μM within ca. 10 h. The oscillatory trend and the rapid decrease in H_2_O_2_ levels suggest that with the nanoparticles, nearly all the generated H_2_O_2_ had been immediately transformed into less stable free radical species, in a process catalyzed by the Fe^2+^ ions released during their rapid dissolution. However, the microparticles gave vastly distinct results: the H_2_O_2_ was generated much more slowly, and gradually accumulated in solution (Figure 3, curve b). This observation suggests a less efficient conversion of H_2_O_2_ into free radicals, which would be consistent with a lower rate of iron delivery to solution than in the case of the nanoparticles. This hypothesis is consistent with PHREEQC calculations [27] of the total iron [Fe^2+^ + Fe^3+^] released (Figure 3, inset) in each case, using the rate expression of Williamson and Rimstidt [28]. The reactive surface was estimated by the geometrical model assuming cubes of 20 nm for nanoparticles and 1.4 μm for microparticles.

**Figure 4:**
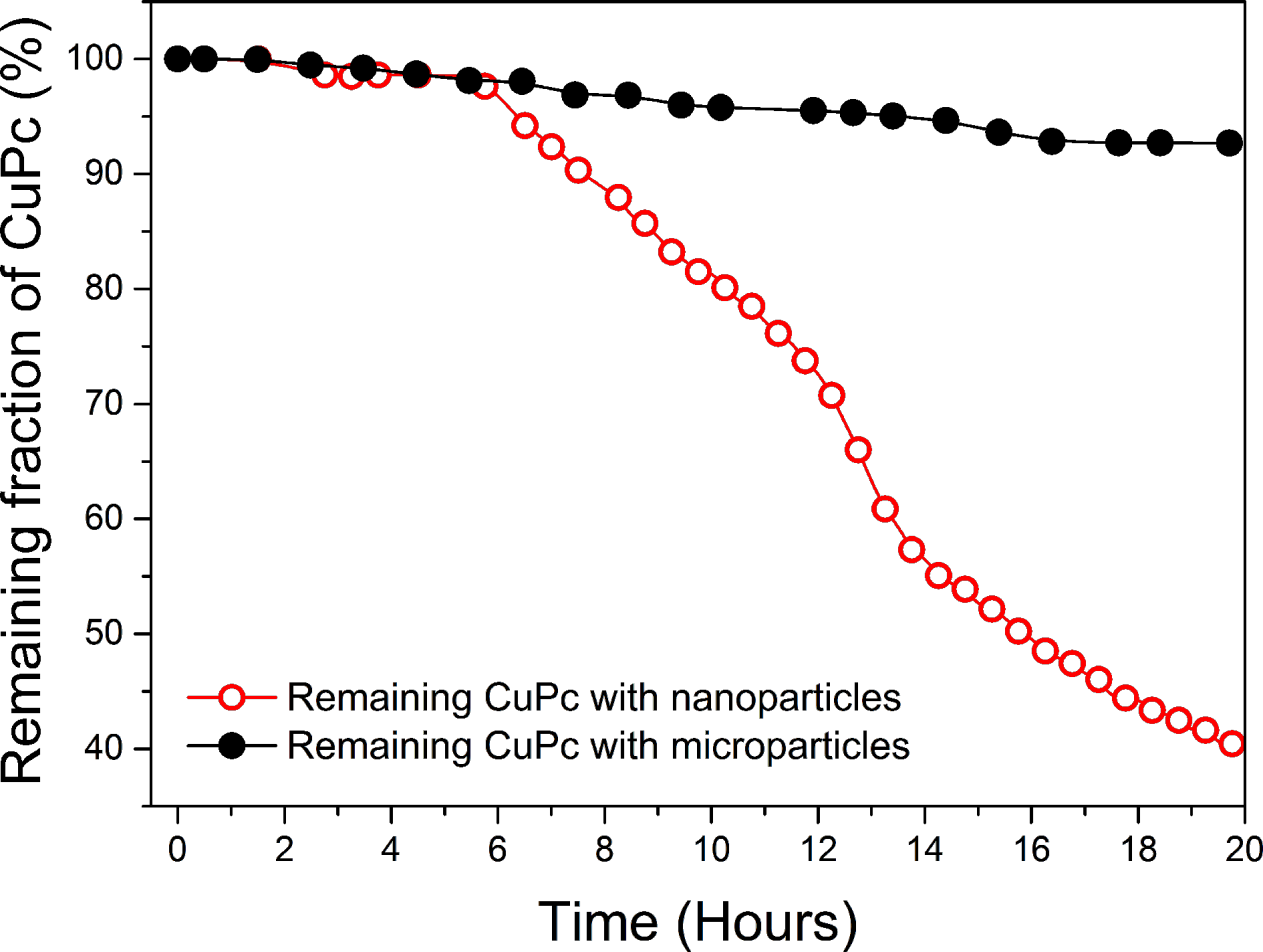
Rate of CuPc decoloration (initial concentration: 0.1 mg/L) in suspensions of pyrite nanoparticles (average size: 20 nm; red circles) or microparticles (average size: 1.4 μm; black circles), at loadings of 0.08 g/L.

The degradation efficiency in each case is shown in Figure 4. During the start-up period (about 5 to 6 h), decomposition was very similar in each reaction, with a rate constant of *k*_1_ = 0.004 h^−1^. Afterwards, the rate constant in the nanoparticle reaction increased to *k*_2_ = 0.07 h^−1^, which in terms of degradation efficiency ([1 − (*C*/*C*_0_)] × 100%) represents a final value ca. 8 times higher than in the microparticle experiments.

#### Effect of pyrite particle loading and phthalocyanine concentration

**Figure 5:**
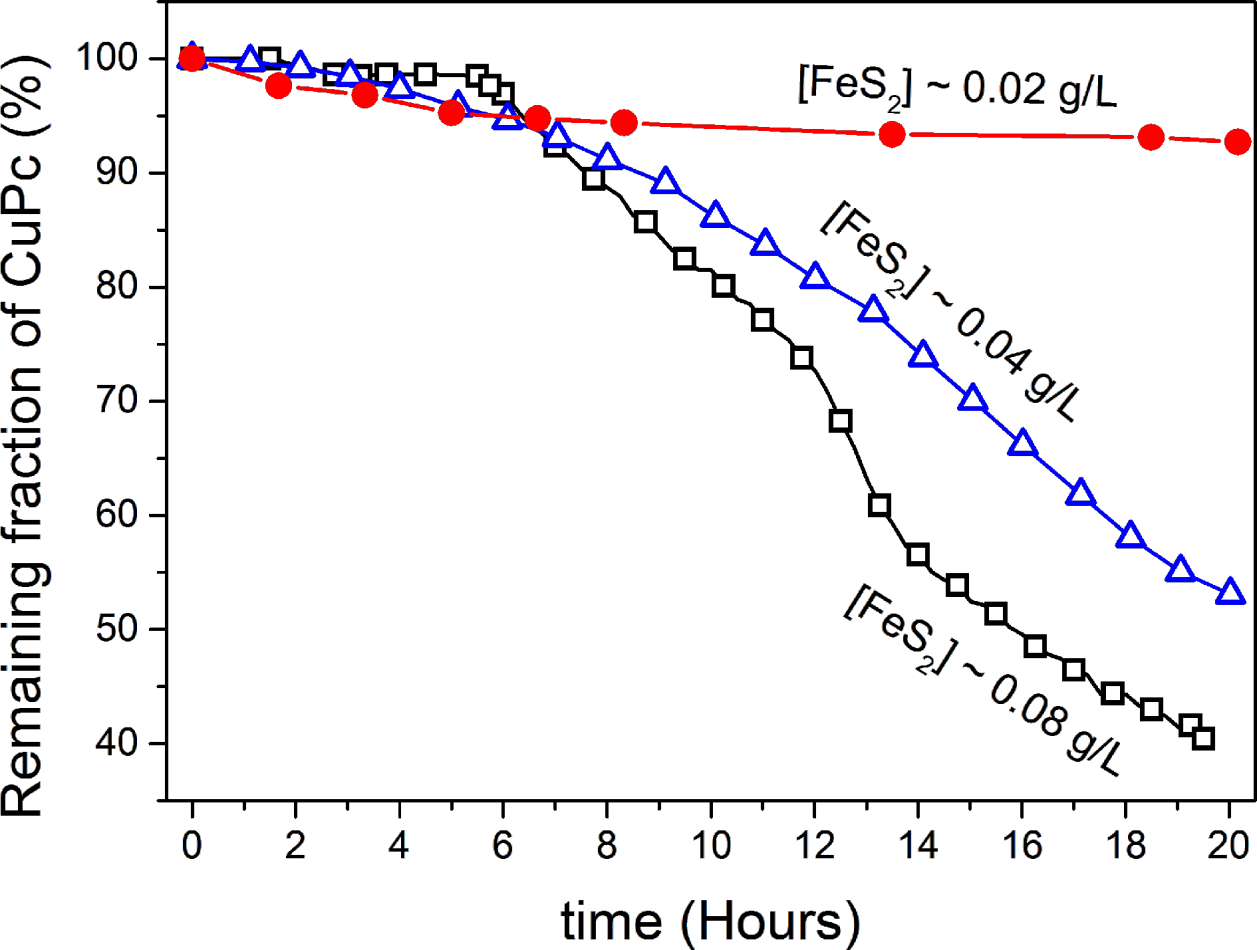
Effect of pyrite nanoparticle loading on the rate of CuPc decoloration. ([CuPc]_0_ = 0.1 mg/L, loadings values are marked in the curves).

The influence of the initial mass of pyrite on the CuPc degradation was assessed by performing experiments with nanoparticles at three different loadings. As shown in Figure 5, even small differences on pyrite loadings prompted changes in the degradation efficiency of CuPc, with the higher loading corresponding to a faster CuPc decomposition (*k*_1_ = 0.07 h^−1^ at 0.08 g/L, *k*_2_ = 0.04 h^−1^ at 0.04 g/L and *k*_3_ = 0.003 h^−1^ at 0.02 g/L) and consequently leading to more efficient degradation (the higher loading experiment was about 8 times more efficient).

**Figure 6:**
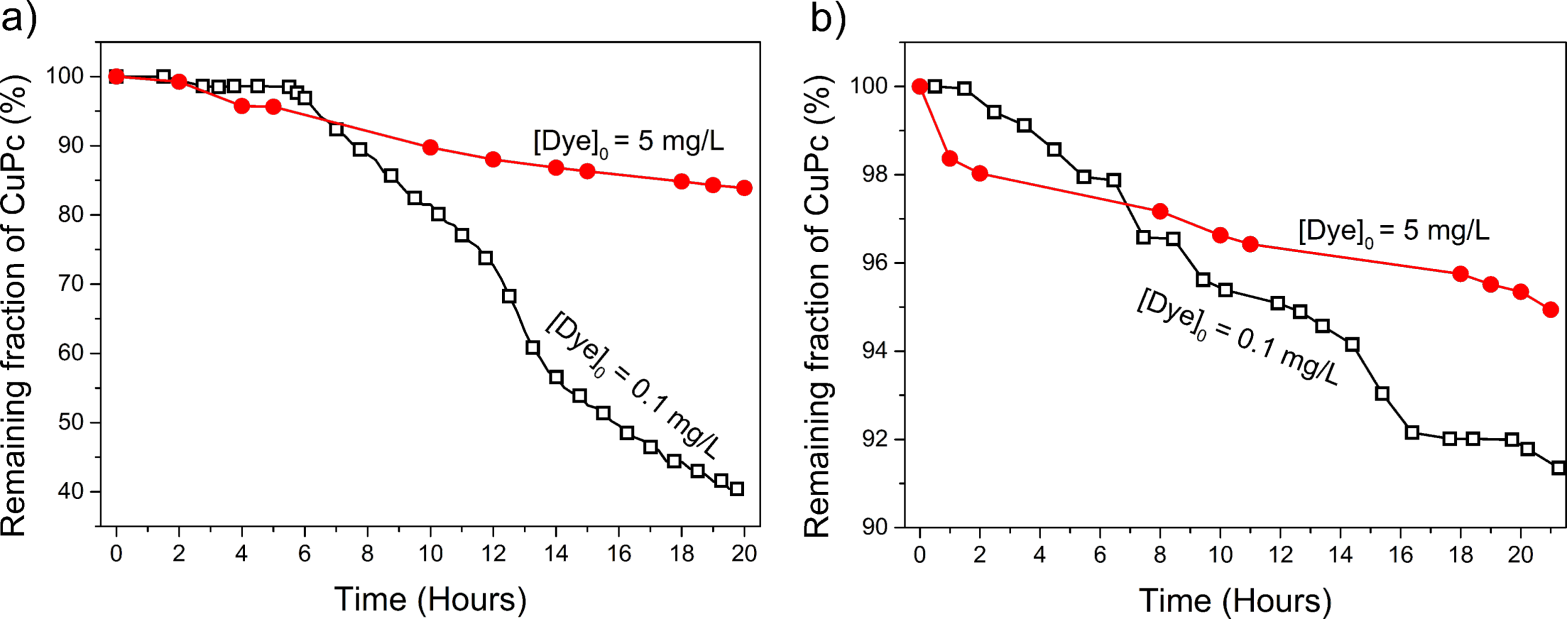
Decoloration rate at different initial concentrations of CuPc in suspensions of pyrite a) nanoparticles or b) microparticles, at loadings of 0.08 g/L.

We also evaluated the influence of the initial concentration of CuPc on its degradation. The results are plotted in Figure 6, in which the curves represent the fraction of remaining contaminant (*C*/*C*_0_) obtained with pyrite nanoparticles (Figure 6a) or microparticles (Figure 6b). In both cases, the degradation proceeded much more quickly at lower levels of dye (nanoparticles: ca. 4 times; microparticles: ca. 2 times), corroborating previous reports of delayed degradation at higher dye concentrations [29]. Nevertheless, experiments with a relatively concentrated dye solution (5 mg/L) and low nanoparticle loading (0.08 g/L) still gave a degradation efficiency of 17% within 20 h.

**Figure 7:**
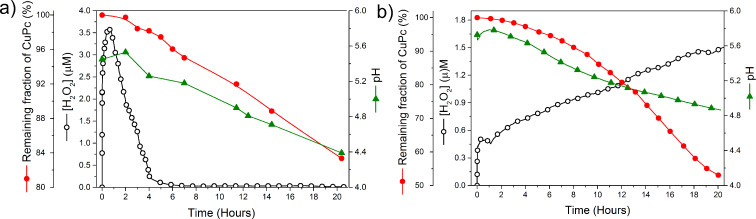
The pH and H_2_O_2_ evolution in CuPc solutions with pyrite nanoparticles (a) [CuPc]_0_ = 5 mg/L at 0.06 g/L loading (b) [CuPc]_0_ = 0.1 mg/L at 0.04 g/L loading.

The use of pyrite nanoparticles for CuPc degradation acidified the system. In the performed experiments, the pH usually attains a final value of the order of 4–5, where Fenton reaction is far more efficient. Consequently, special pH control is not required. This it shown in Figure 7, for experiments performed with two different concentration values of dye and pyrite nanoparticle loads.

**Figure 8:**
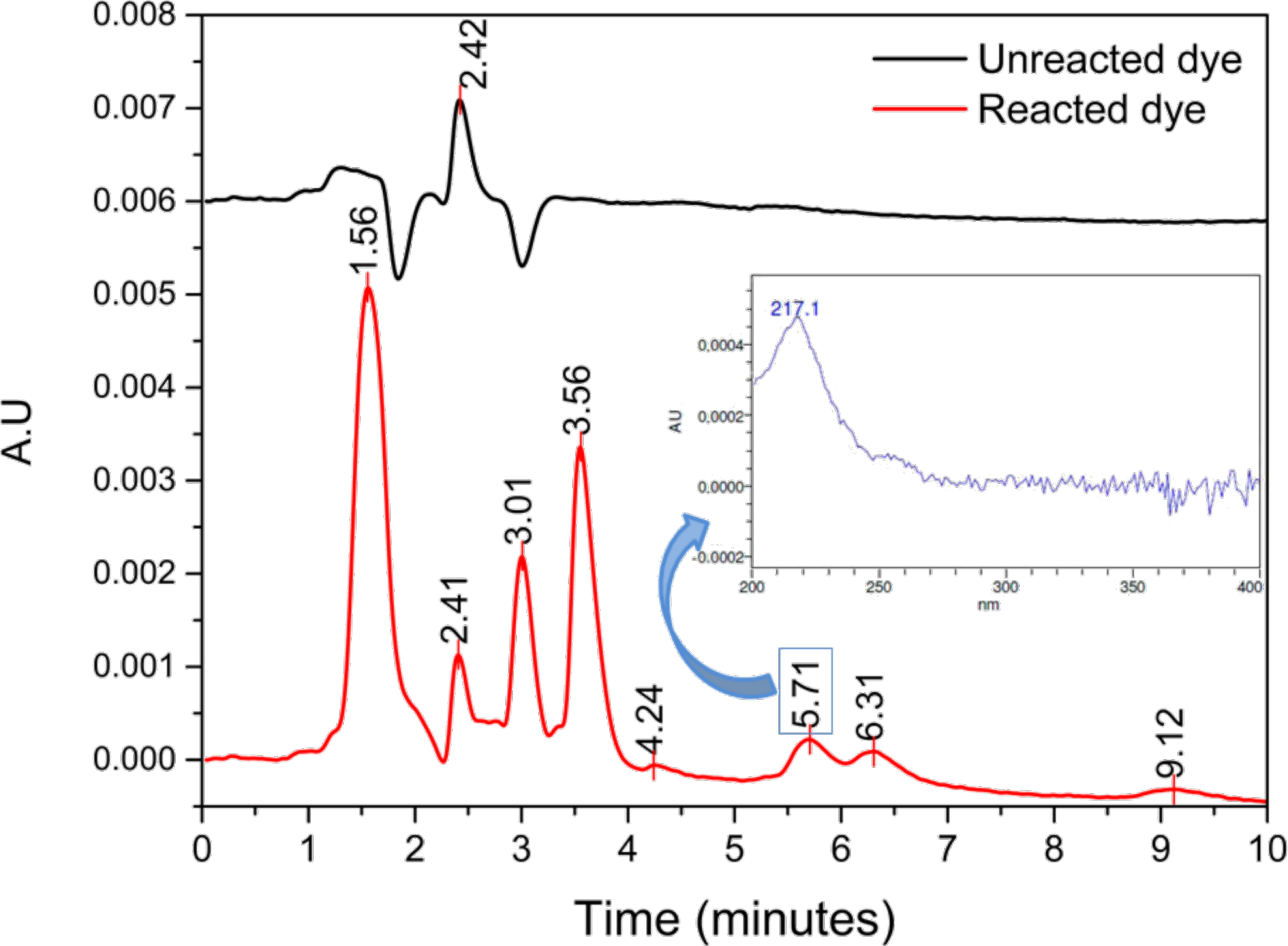
a) HPLC Chromatograms (UV detection: 219 nm) of untreated dye, and of dye treated with suspensions of nanoparticles for 27 h. b) UV-vis spectrum of the peak at 5.7 minutes (λ_max_: 217 nm).

We used high performance liquid chromatography (HPLC) to identify byproducts generated during degradation. The chromatogram of unreacted dye was employed as control (Figure 8) and showed a single peak in the UV region with a retention time of 2.42 minutes. After 27 h of reaction with nanoparticles, the chromatogram exhibited seven new peaks (retention times: 1.56, 3.01, 3.56, 4.24, 5.71, 6.31 and 9.12 minutes, respectively) and an appreciable decrease in the intensity of peak corresponding to the dye (at 2.42 minutes). The peak at 5.71 minutes, which is associated with phthalamines, a diagnostic species for the oxidative destruction of phthalocyanines [30], showed a λ_max_ of 217 nm.

## Discussion

**Figure 9:**
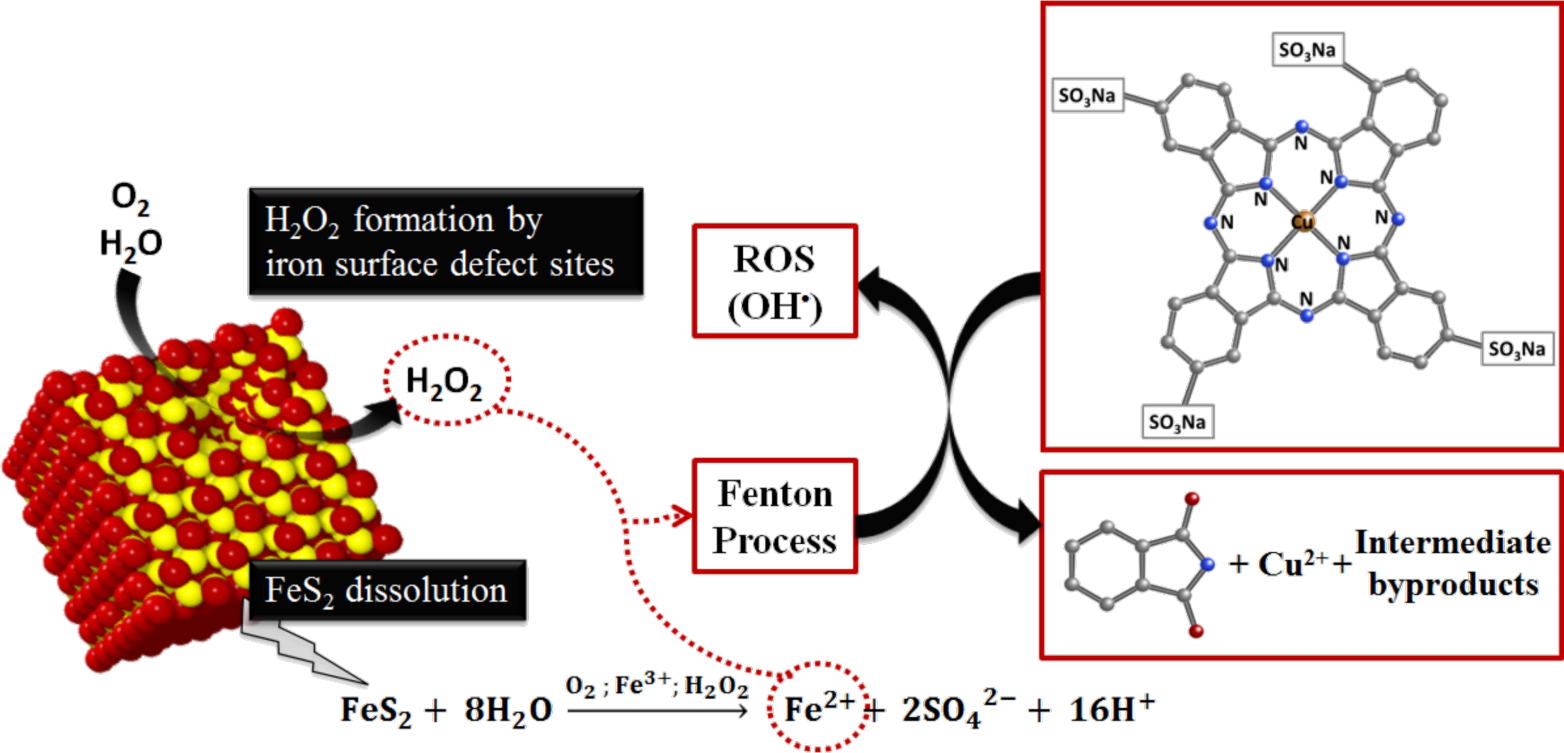
Proposed reaction mechanisms for the generation of H_2_O_2_ and for the oxidative degradation of CuPc by OH^•^.

There have been previous reports on the surface generation of H_2_O_2_ upon oxidative dissolution of pyrite [7–8,10,12–14]. This reactivity, coupled with the iron delivery and the decrease in pH that occur upon dissolution of pyrite, are the reasons that this mineral is ideal for use in wastewater treatments. Furthermore, in the present work, we have demonstrated the efficiency of pyrite at breaking down the ring of CuPc, which we confirmed through the HPLC identification of sulfophthalimide, the most common oxidative byproduct of this dye [30]. Practical interest in pyrite as a Fenton-type reagent depends on its capability to efficiently and sustainably generate H_2_O_2_ for oxidative degradation of contaminants. Figure 9 summarizes the proposed reaction mechanisms which are involved in the H_2_O_2_ generation and in the subsequent degradation of CuPc by the hydroxyl radical [29].

Although the mechanism of H_2_O_2_ generation remains controversial [7,10–15,31–32], researchers agree that this product is afforded by reaction of iron defect sites at the pyrite surface with adsorbed oxygen and water, according to Equation 1 and Equation 2, below. This chemistry involves the intermediate generation of O_2_^•−^ from dissociative adsorption of O_2(g)_ at the pyrite surface:

[1]
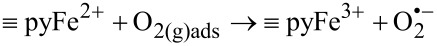


[2]
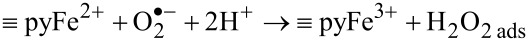


The adsorbed H_2_O_2_ is then released into the solution. Simultaneously, the amount of Fe^2+^ required for the Fenton reaction to occur is supplied to the solution by oxidative dissolution of pyrite in the presence of O_2_(aq), according to Equation 3:

[3]
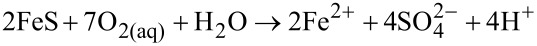


At this moment, Fe^2+^ starts to catalyze the decomposition of H_2_O_2_ into OH^•^ and other reactive oxygen species involved in the oxidation of organics pollutants, according to the Fenton chain-reaction sequence, described by Equations 4 to 7, below [33].

[4]
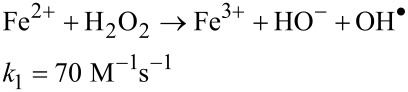


[5]
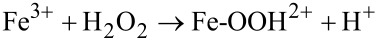


[6]
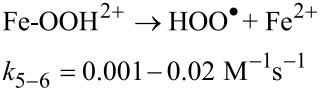


[7]
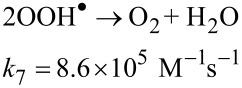


In this sequence, Equation 5 and Equation 6 are the slowest and diminish the recycling between Fe^2+^ and Fe^3+^. Consequently, they form the rate-limiting step in the generation of free radical species and decrease the efficiency of the oxidative degradation of contaminants. When pyrite is used as iron source for heterogeneous catalysis, an additional mechanism for the Fe^2+^ regeneration occurs through Equation 8:

[8]
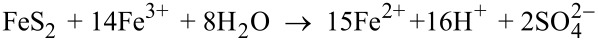


This drives the oxidation of pyrite by Fe^3+^, thereby releasing Fe^2+^ and consequently, accelerating the degradation of H_2_O_2_ through Equation 4. As shown above, Equation 3 and Equation 8 both release protons, promoting acidification simultaneously to the delivery of iron into solution.

The rate expressions associated to Equation 3 and Equation 8 are, respectively [28]:

[9]
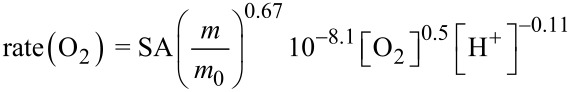


[10]
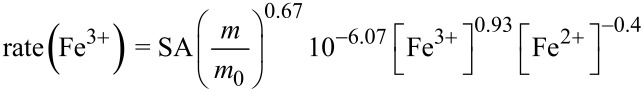


Both equations depend on the reactive surface, SA (1/dm). Since H_2_O_2_ is also formed by reactions at the surface, the dye degradation efficiency is expected to increase with a greater surface area (i.e., smaller particles should give better results). We assessed this hypothesis by performing batch experiments to compare the degradation behavior of nanoparticles with that of microparticles (Figure 3 and Figure 4).

As expected, the nanoparticles gave strikingly better results than the microparticles. At low loading (0.08 mg/L) and 20 h reaction time, the former enabled 60% CuPc removal, whereas the latter enabled only 7% removal. As such, the H_2_O_2_ was consumed far more quickly when nanoparticles were used. This can be explained by the fact that the greater surface area of the nanoparticles not only provides more sites for H_2_O_2_ generation, but also leads to faster oxidative dissolution of the pyrite itself, as this rate is proportional (in mass terms) to the reactive surface area. Thus, the nanoparticles rapidly supply the solution with iron, which induces the rapid transformation of H_2_O_2_ into OH^•^ radicals, according to the Fenton reaction scheme. The dye concentration was indirectly proportional to the degradation rate (Figure 6), indicating that the best catalytic activities were reached by using dilution procedures that provide low concentrations of contaminant. Moreover, the system does not require a pH control to degrade CuPc (Figure 7).

## Conclusion

We evaluated the use of synthetic pyrite nanoparticles and naturally derived pyrite microparticles for Fenton-like oxidative degradation of the dye CuPc as representative industrial contaminant. Since pyrite spontaneously and sustainably releases H_2_O_2_ upon surface reaction with adsorbed O_2(g)_ and H_2_O, it might prove invaluable for Fenton-like treatment of wastewater, obviating the need for external addition of H_2_O_2_. Furthermore, dissolution of pyrite in water promotes the recycling of Fe^2+^ into Fe^3+^ and vice versa, triggering Fenton production of HO^•^, the primary species responsible for oxidative degradation of the pollutant. Our results with the synthetic pyrite nanoparticles demonstrate that H_2_O_2_ is indeed generated by iron disulfide, in the absence of trace compounds founds in natural pyrite.

Our kinetics analysis showed that the pyrite nanoparticles enabled a ca. 8 times greater efficiency of pollutant removal than did the microparticles. The use of synthetic pyrite nanoparticles avoids the dangers of heavy metal release that often occurs upon dissolution of naturally occurring (i.e., mineral derived) pyrites. Furthermore, the low required loadings of these nanoparticles make this procedure even more environmentally friendly. Lastly, since the process does not require UV-illumination or pH constraints, it may serve as a cheap alternative to conventional Fenton approaches for the selective oxidation of pollutant dyes.

## Experimental

### Chemicals

Copper phthalocyanine-3, 4´, 4´´, 4´´´- tetrasulfonic acid tetrasodium salt (85%), oleylamine (OA, 70%), sulfur (99.99%) and toluene (99.8% anhydrous) were purchased from Sigma Aldrich. Ferrous chloride tetrahydrate (FeCl_2_·4H_2_O, 99%) was purchased from Fluka. Absolute ethanol was purchased from Quimivita. All chemicals were used as received without any further purification. Aqueous suspensions of pyrite were prepared using deionized water (resistivity: ca. 18 MΩ·cm) purified in a Milli-Q system at an initial pH of about 5.5.

#### Synthesis of pyrite nanoparticles

Pyrite nanocrystals were synthesized by the hot injection method [26,34]. The experiments were performed in a three-neck flask connected to a reflux condenser. The device was heated by an electric mantle temperature-probe controlled. Sulfur and FeCl_2_·4H_2_O were used as starting materials. Briefly, 0.4 mmol of FeCl_2_·4H_2_O were dehydrated and dissolved in 6 mL of OA under N_2_ atmosphere. The resulting solution was maintained at 100 °C for 1 h, until an Fe–OA complex was formed. A solution of 2.4 mmol of sulfur in 6 mL of OA (to achieve an Fe/S molar ratio of 1:6) was injected, heated to 220 °C and allowed to react for 20 min. The mixed solution was cooled to room temperature. Nanocrystals were dispersed and separated by centrifugation and re-dissolving with several aliquots of a 1:1 toluene/ethanol solution.

#### Preparation of pyrite microparticles

Natural pyrite cubes (Logroño, Spain) were milled using a diamond disk and sieved (63 μm) to obtain pyrite powder. The resulting particles presented an average diameter of 1.4 μm (laser diffraction particle size analyzer, LS13320) and a specific surface area (BET) of 1.46 m^2^/g. Prior to use, the pyrite samples were cleaned by sonication in ethanol (96%), HCl (0.25 M) and deoxygenated water, and then dried under vacuum, purged with N_2_ and finally, stored in a glove box (N_2_ atmosphere) until use.

#### Characterization

The nanoparticles were characterized by high-resolution transmission electron microscopy (HR-TEM), selected area electron diffraction (SAED) and X-ray diffraction (XRD). The TEM studies were done on a JEOL JEM-3011 microscope with accelerating voltage of 200 kV. The XRD analysis of the nanoparticles and the microparticles was done on a Philips diffractometer with a graphite monochromator and Cu Kα radiation (1.54 Å). Indexing of pyrite reflections were done using the JCPDS 00-042-1340 card (FeS_2_ [pyrite]). Further, Rietveld refinements were performed to rule out the presence of other crystalline iron sulfides (e.g., marcasite, pyrrhotite and troilite) and to calculate the average values of crystallite size and microstrain (according to the Scherrer method [35]).

#### Experimental set-up

Kinetic experiments were conducted in stirred glass reactors under ambient conditions (i.e., open to atmosphere and at room temperature [22 ± 2 °C]; K-type thermocouple). Pyrite powder was deposited onto silicone strips as a thin film of particles, and the strips were then adhered to the inner reactor walls. The pH was monitored by a glass pH-meter (Vernier FPH-BTA) with an Ag/AgCl reference electrode.

The temporal change in the H_2_O_2_ concentration in solution was monitored by an amperometric microsensor (ISO-HPO-100, World Precision Instruments, Inc.). These sensors contain a flexible, activated carbon-fiber sensing electrode coated with a proprietary membrane that enhances the low detection limit (LDL) of H_2_O_2_ to a value of 10 nM (ten times lower than in the bare Pt electrode; LDL: 0.1 μM) with a response time of less than 5 s. The signal was amplified with a picoamperemeter (Apollo 4000 Free Radical Analyzer, World Precision Instruments). Measurements were taken by using a polarization voltage of 0.4 V versus an Ag/AgCl reference electrode.

**Figure 10:**
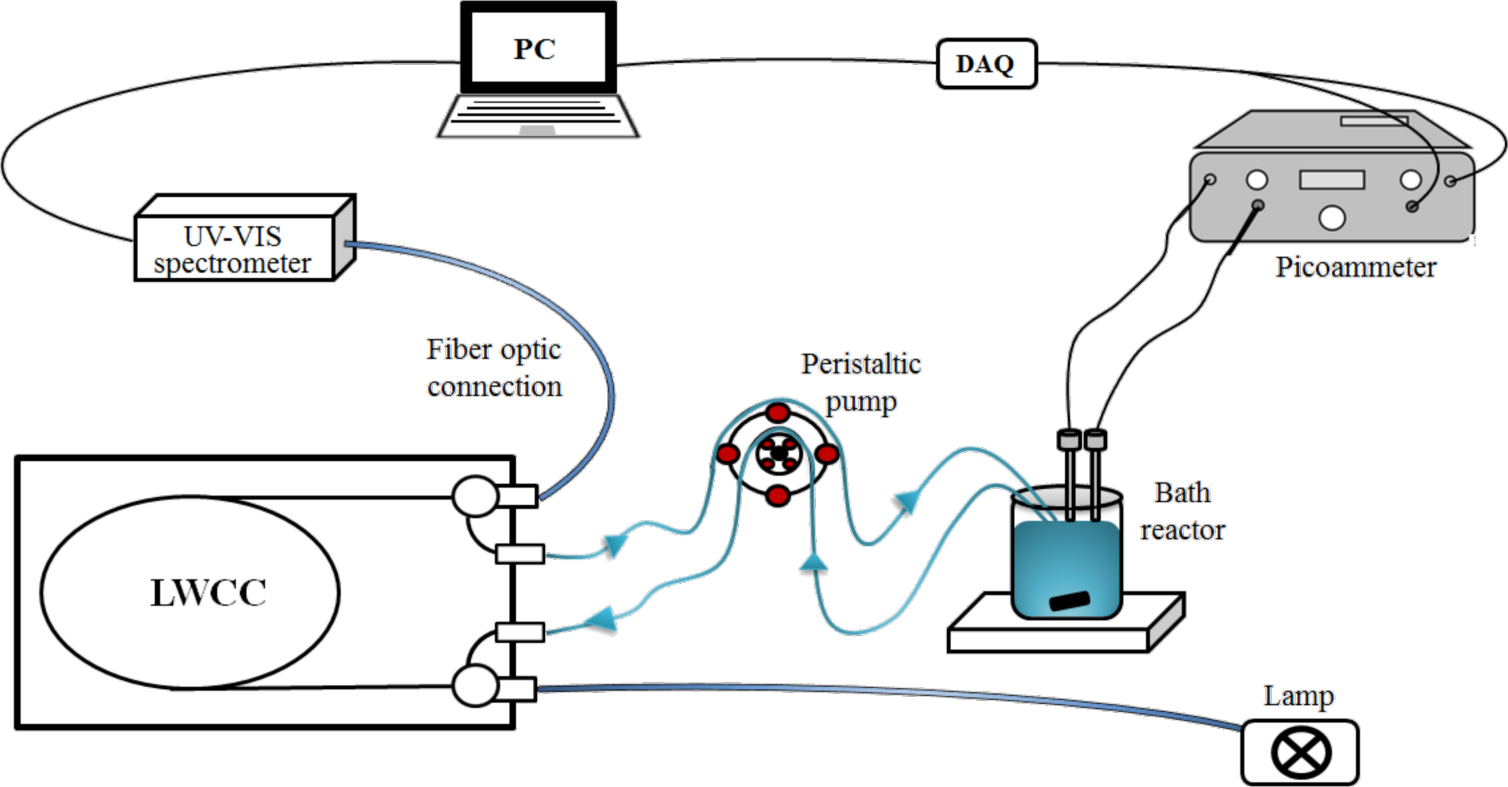
Experimental set-up using a liquid waveguide capillary flow cell (LWCC).

Simultaneous to H_2_O_2_ generation, the decoloration (degradation) of CuPc was monitored by tracking the absorbance at 630 nm, using fiber optic UV–vis spectrometry (Black-comet, Stellarnet). A liquid waveguide capillary flow cell (LWCC; path length: 250 cm; WPI), was connected to the batch reactor by a peristaltic pump (masterflex pump system, Cole-Parner Instrument Co; see Figure 10). Alternatively, when the initial dye concentration was too high (i.e. when it led to saturation in the Vis spectra obtained with the LWCC), a standard quartz cuvette (path length: 1 cm) was used and the decoloration of the dye was done measuring aliquots at different times of the process.

The degradation of CuPc was analyzed by high performance liquid chromatography (HPLC; Waters Alliance 2975 equipped with a Waters 996 Photodiode Array Detector; UV–vis detection), using a C8 column (Waters Symmetry: 150 × 4.6 mm, 3.5 μm). The mobile phases were 0.1% aq phosphoric acid (A), and acetonitrile (B), run in a linear gradient (80:20, v/v) at a flow rate of 1 mL/min.
